# Metformin combined with p38 MAPK inhibitor improves cisplatin sensitivity in cisplatin-resistant ovarian cancer

**DOI:** 10.3892/mmr.2014.2490

**Published:** 2014-08-14

**Authors:** YA XIE, ZHENG PENG, MINGXING SHI, MEI JI, HONGJUN GUO, HUIRONG SHI

**Affiliations:** Department of Obstetrics and Gynecology, The First Affiliated Hospital of Zhengzhou University, Zhengzhou, Henan 450052, P.R. China

**Keywords:** metformin, cisplatin-resistant ovarian cancer, p38 MAPK, cell proliferation

## Abstract

The aim of the present study was to determine the effects of metformin, combined with a p38 mitogen-activated protein kinase (MAPK) inhibitor, on the sensitivity of cisplatin-resistant ovarian cancer to cisplatin. The expression and distribution of phosphorylated p38 MAPK (P-p38 MAPK) was confirmed in drug-resistant and primary ovarian cancer tissues by immunohistochemistry and western blotting. A bromodeoxyuridine ELISA kit was used to analyze the effects of metformin, SB203580, a p38 MAPK inhibitor, and metformin combined with SB203580, on the cell proliferation of SKOV3/DDP cisplatin-resistant ovarian cancer cells. The protein expression of P-p38 MAPK was significantly higher in cisplatin-resistant ovarian cancer, as compared with the primary ovarian cancer tissues. Metformin combined with SB203580 significantly enhanced the sensitivity of SKOV3/DDP cells to cisplatin. In conclusion, the p38 MAPK signaling pathway may be associated with cisplatin-resistant ovarian cancer. Metformin, combined with the p38 MAPK inhibitor, significantly increased the sensitivity of SKOV3/DDP cells to cisplatin treatment.

## Introduction

Ovarian cancer is a gynecological malignancy with the highest known risk of mortality (1). It has been reported that ~70% of patients, at initial presentation, are diagnosed with advanced ovarian cancer (International Federation of Gynecology and Obstetrics stage III-IV) (2). The standard treatment for ovarian cancer is up-front cytoreductive surgery, followed by platinum and paclitaxel chemotherapy every three weeks (3). Chemotherapy-resistant advanced ovarian cancer is associated with a five year survival rate of <30% (4). Disease recurrence within six months is classed as resistance to treatment (5), with relapse occurring in nearly all of the patients tolerant to chemotherapy. There is currently no clinically effective treatment for recurrent ovarian cancer. It is therefore important to find a novel strategy to reverse chemoresistance and improve the survival rate of patients with ovarian cancer.

Metformin is an oral anti-diabetic drug in the biguanide class that is used in the treatment of type II diabetes. Previously, two large epidemiological studies demonstrated that long-term use of metformin reduced the risk of ovarian cancer occurrence (6). Furthermore, in patients with ovarian cancer combined with diabetes, metformin treatment was shown to significantly increase progression-free survival, as compared with those who were not administered with metformin (7). Previously, metformin was found to inhibit cell growth in ovarian cancer *in vitro* (8), although the mechanism for this is unclear. Furthermore, metformin significantly enhanced the effects of cisplatin, paclitaxel and doxorubicin in the inhibition of tumor cell growth, reduced the required dose of doxorubicin, and prolonged disease remission in lung, breast and prostate cancer nude mice models (9–11). These results identify metformin as a potential regulator of tumor cell sensitivity to chemotherapeutic drugs. However, the mechanism remains unclear.

Cell damage and chemoresistance are mediated primarily through the mitogen-activated protein kinase (MAPK) and phosphoinositide kinase-3-threonine protein kinase B signaling pathways (8). The signaling pathways mediated by the MAPK family include p38 MAPK, extracellular signal-regulated kinase (ERK), c-jun N-terminal kinase and other subfamilies; of these pathways the p38 MAPK and ERK1/2 are considered to be the most important. Phosphorylated MAPK subsequently phosphorylates the B cell lymphoma-2 (Bcl-2) and Bcl-2-associated death proteins, which have been shown to weaken the effects of platinum and taxane in tumor cell apoptosis and increase cancer resistance to chemotherapeutic drugs (12,13). The MAPK signaling pathway has an important role in cell proliferation, apoptosis and chemoresistance in a variety of malignant tumors, including ovarian cancer (14,15).

In the present study, MAPK pathway activation was investigated in paclitaxel and platinum-resistant ovarian carcinoma specimens. The cell proliferation of SKOV3/DDP cisplatin-resistant ovarian cancer cells was determined using a bromodeoxyuridine (BrdU) ELISA kit. The effects of metformin on cell proliferation, irrespective of the presence of a p38 MAPK signaling pathway inhibitor, were confirmed in the SKOV3/DDP cell line. The expression of phosphorylated p38 MAPK (P-p MAPK) was determined in both drug-resistant and primary ovarian cancer tissues. The effects of metformin, both alone and in combination with a p38 MAPK inhibitor, were observed on the reversal of ovarian cancer cisplatin-resistance in SKOV3/DDP cells. In addition, the present study investigated the therapeutic mechanisms of metformin in drug-resistant ovarian cancer, in an effort to develop novel clinical strategies against recurrent ovarian cancer.

## Materials and Methods

### Materials

A total of 20 pairs of epithelial ovarian cancer (EOC) tissue samples were collected from the archives of the Department of Gynecology of the First Affiliated Hospital of Zhengzhou University (Zhengzhou, China), between July 2012 and May 2013. The tissue samples were obtained from patients who had been treated with cytoreductive surgery and standard chemotherapy, but had relapsed following treatment. The criteria for enrollment to the study were as follows: Complete medical records, confirmed pathological diagnosis, and disease recurrence following standard chemotherapy treatment. The tissue samples of both the primary and recurrent cancers were collected. The tissue samples of the control group were collected from patients with ovarian cancer, following cytoreductive surgery, but not chemotherapy. All specimens were collected within 30 min of excision from the patient, and stored at −80°C until further use. The specimens were collected after obtaining the informed consent from the patients. The study was approved by the Ethics Committee of the First Affiliated Hospital of Zhengzhou University.

### Cell lines and reagents

SKOV3/DDP, adherent and moderately/well differentiated, cisplatin-resistant cells of human ovarian serous cystadenocarcinoma, were maintained in phenol red RPMI-1640 medium, supplemented with 10% fetal bovine serum (FBS) at 37°C in 5% CO_2_. The cell cultures were routinely passaged every 3–5 days. The rabbit anti-human polyclonal antibodies: p38 MAPK, P-p38 MAPK, and GAPDH were purchased from Cell Signaling Technology Inc. (Danvers, MA, USA); metformin and the p38 MAPK inhibitor SB203580 were purchased from Sigma-Aldrich (St. Louis, MO, USA); RPMI-1640 culture medium and FBS were purchased from Gibco-BRL (Carlsbad, CA, USA); and the BrdU ELISA kit was purchased from Roche Diagnostics GmbH (Mannheim, Germany).

### Immunohistochemical staining

The paraffin-embedded blocks of primary and recurrent ovarian cancer specimens were sectioned at 4 μm thickness and mounted onto slides. The sections were fixed with 10% paraformaldehyde, and the immunohistochemical streptavidin peroxidase-conjugated method was adopted. The tissue sections were processed in strict accordance with the manufacturer’s instructions. Phosphate-buffered saline (PBS; Thermo Fisher Scientific, Boston, MT, USA) was used, instead of primary antibody, as a negative control and a known positive plate was used as a positive control. The distribution of P-p38 MAPK in the cancer tissue was visualized and quantified to the average gray value. Gray-scale images were examined, depending on the size and shape of the tissue.

### Western blotting

Protein was extracted from the ovarian cancer tissues using radioimmunoprecipitation assay buffer, containing 1% nonyl phenoxypolyethoxylethanol-40, 0.5 sodium deoxycholate and 0.1% sodium dodecyl sulfate. A total of 20 μg protein extract was separated by 10% SDS-PAGE, and electrotransferred onto a nitrocellulose membrane. Subsequently, the membrane was blocked with 5% nonfat dry milk and 0.1% Tween^®^ 20 for 1 h at room temperature with constant agitation, followed by incubation with a primary antibody (1:1,000 dilution) overnight at 4°C. The membranes were washed three times each, for 5 min, with PBST (PBS with Tween), and the membrane was incubated with a secondary horseradish peroxidase-linked antibody (1:2,000 dilution) for 2 h. The membrane was then washed a further three times with PBST, and the bands were visualized by enhanced chemiluminescence, according to the manufacturer’s instructions (Pierce Biotechnology Inc., Rockford, IL, USA). The relative protein expression was normalized to GAPDH (1:1,000 dilution), and expressed as a ratio to the control subjects. The protein bands, including GAPDH, were quantified by densitometry using the Quantity One^®^ imaging program (Bio-Rad, Hercules, CA, USA).

### Cell proliferation studies

Cell proliferation assays were performed using the BrdU ELISA kit (Roche Diagnostics GmbH). The SKOV3/DDP cells were plated into 96-well plates at a concentration of 1×10^4^ cells/well for 24 h. The cells were subsequently serum-starved for an additional 24 h, and treated with different concentrations of metformin and 5 μM SB203580 for 72 h. The effects of metformin and SB203580 were calculated as a percentage of the control cell growth, obtained from PBS or dimethyl sulfoxide-treated cells grown in the same 96-well plates. The assays were performed under serum-free conditions. DNA synthesis was monitored based on the incorporation of BrdU into the DNA, which was detected by immunoassay, according to the manufacturer’s instructions. Following incubation, the cells were re-incubated with 10 μl/well BrdU labeling solution for an additional 2 h at 37°C. The labeling medium was removed, 200 μl/well FixDenat (Selleck, Houston, TX, USA) was added, and the cells were incubated for 30 min at 20°C. The FixDenat solution was subsequently removed. The cells were incubated with 100 μl/well anti-BrdU peroxidase working solution for 90 min at 20°C. The antibody conjugate was removed and the cells were rinsed three times with washing solution. Following the removal of the washing solution, and the addition of 100 μl/well substrate solution, the cells were incubated at 20°C for 20 min, followed by the addition of 25 μl of 1 M H_2_SO_4_. The cells were incubated for 1 min with agitation at 300 rpm, and the absorbance of the samples was measured in an ELISA reader (Thermo Fisher Scientific) at 450 nm, with a reference wavelength of 690 nm. Each experiment was performed in triplicate, and repeated three times in order to assess the consistency of the results. The results were also compared using the BrdU technique with an MTT assay, which confirmed the validity of the findings (data not shown).

### Statistical analyses

All statistical analyses were performed using SPSS version 13.0 software (SPSS Inc., Chicago, IL, USA). The data between the two groups were analyzed by a Student’s t-test. The data between multiple groups were analyzed by a one-way analysis of variance. A value of P<0.05 was considered to indicate a statistically significant difference.

## Results

### Expression of P-p38 MAPK protein in drug-resistant and primary ovarian cancer tissues

#### Immunohistochemistry

**Figure 1:**
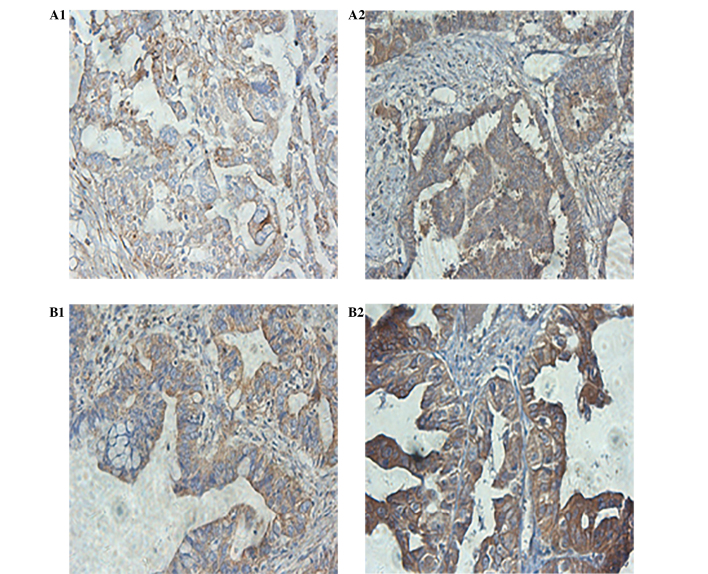
Expression and localization of phosphorylated-p38 mitogen activated protein kinase (P-p38 MAPK) protein in drug-resistant and primary ovarian cancer tissues visualized using streptavidin peroxidase conjugated immunohistochemistry (magnification, ×400). (A1, B1) The relative protein expression of P-p38 MAPK was shown to be reduced in primary ovarian cancer tissues, as compared with (A2, B2) the expression of P-p38 MAPK in drug-resistant ovarian cancer tissues. The cells showing brown granules in the cytoplasm and nucleus were considered to be positively stained, and the percentage of positively stained cells was normalized to the average gray value. The P-p38 MAPK positive signals were observed to be mainly located in the cytoplasm, with few positive signals observed in the nuclei.

P-p38 MAPK protein was observed to be mainly distributed in the cytoplasm, and the nuclei of the cells of both the drug-resistant, and primary ovarian cancer tissues (Fig. 1). The relative expression in the drug-resistant ovarian cancer tissues was significantly increased, as compared with the primary ovarian cancer tissues (P<0.05). The difference in the average gray value of the P-p38 MAPK expression was significantly different between the drug-resistant and the primary ovarian cancer tissues (P<0.05).

#### Western blotting

**Figure 2:**
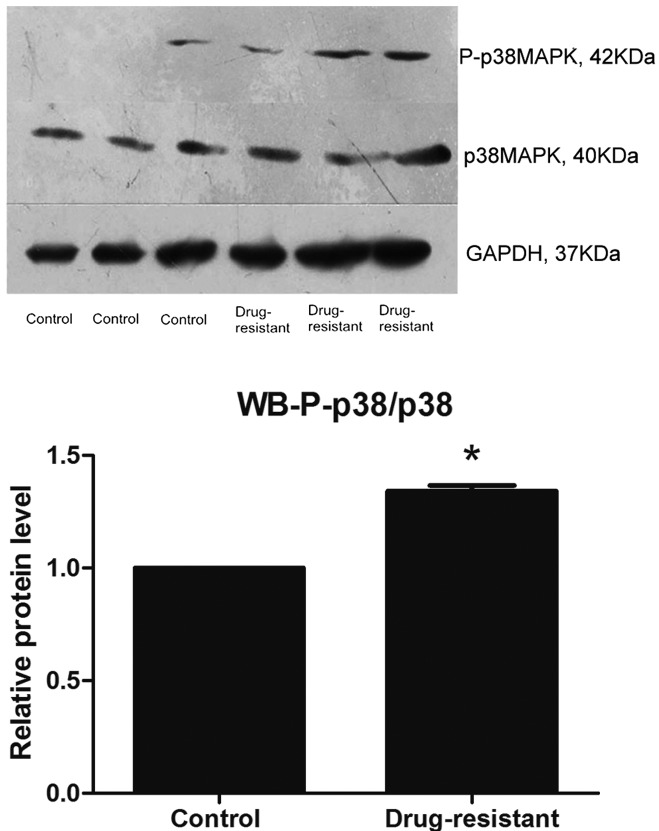
Expression of phosphorylated (P)-p38 mitogen activated protein kinase (MAPK) in chemotherapy-resistant and primary epithelial ovarian cancer tissues. The control group consisted of ovarian cancer tissues following initial cytoreductive surgery. The drug-resistant group was composed of chemotherapy-resistant epithelial ovarian cancer tissues. The levels of P-p38 MAPK and p38 MAPK proteins were detected by western blotting. GAPDH was used as an internal loading control.

The relative expression of P-p38 MAPK protein in chemotherapy-resistant EOC tissues was significantly increased, as compared with the primary ovarian cancer tissues (Fig. 2), as determined by western blotting.

#### Effects of p38 MAPK inhibitor on the expression of p38 MAPK and P-p38 MAPK protein in SKOV3/DDP cells

**Figure 3:**
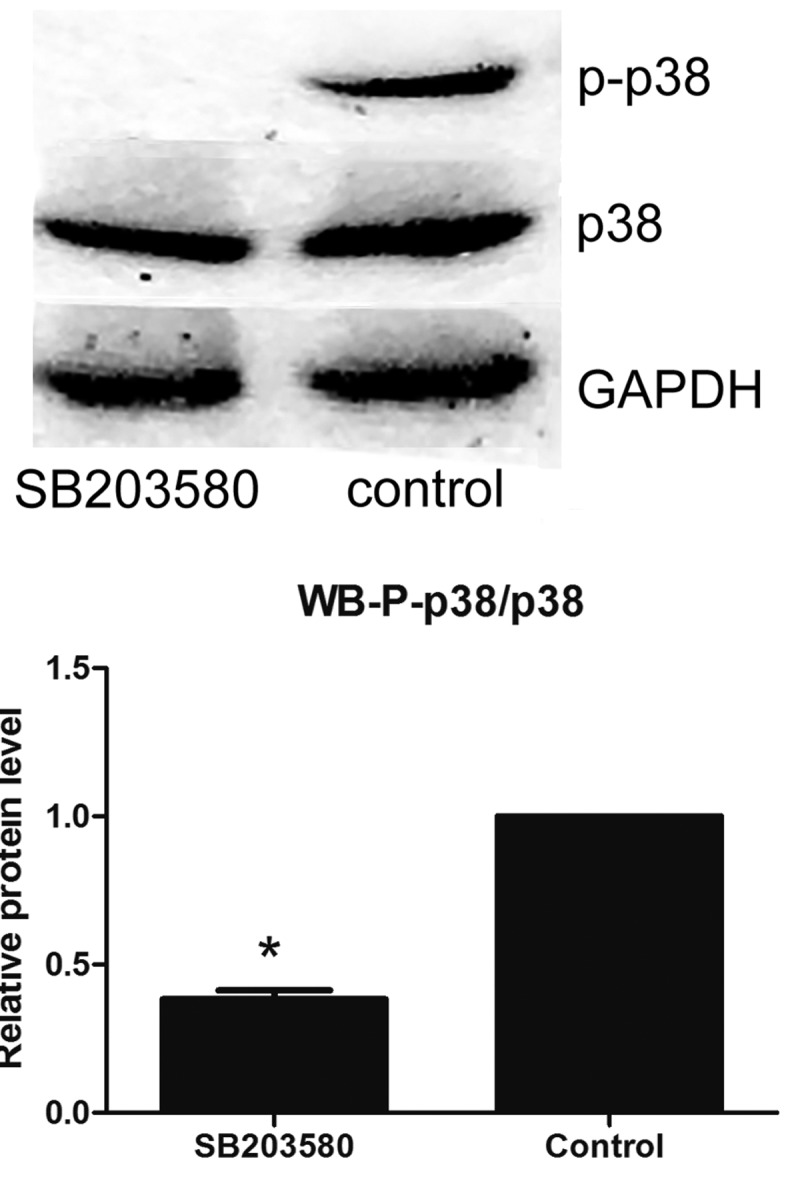
Western blot analysis of phosphorylated (P)-p38 mitogen activated protein kinase (MAPK) and p38 MAPK protein expression from SKOV3/DDP cisplatin-resistant ovarian cancer cells following treatment with p38 MAPK inhibitor SB203580 for 72 h. GAPDH was used as an internal loading control.

Following treatment with the p38 MAPK inhibitor SB203580, the relative expression of P-p38 MAPK protein in SKOV3/DDP cells was shown to be significantly reduced, as compared with the control (Fig. 3).

#### Effect of metformin on the proliferation of endometrial cancer cells

**Figure 4:**
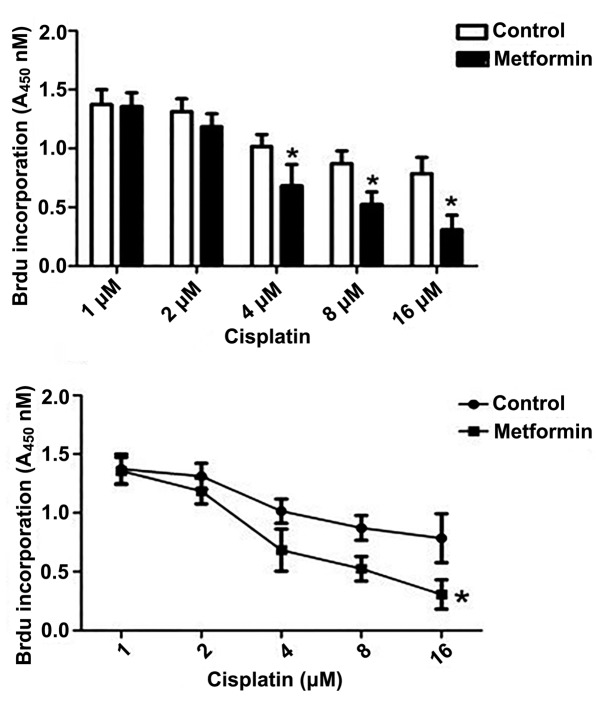
Effects of metformin on SKOV3/DDP cisplatin-resistant ovarian cancer cell proliferation. SKOV3-DDP cells were treated with different concentrations of cisplatin and metformin for 72 h, followed by detection of cell proliferation levels using a bromodeoxyuridine (BrdU) ELISA kit. The results represent the means ± standard error of triplicate samples. Three independent experiments were performed. ^*^P<0.05, one-way analysis of variance.

Cisplatin was shown to markedly inhibit the proliferation of SKOV3/DDP cells, as determined by the BrdU ELISA assay. The most significant effect was observed when the cells were treated with 1 mM metformin (P<0.05) (Fig. 4).

#### Metformin combined with SB203580 significantly improves the sensitivity of SKOV3/DDP cells to cisplatin treatment

**Figure 5:**
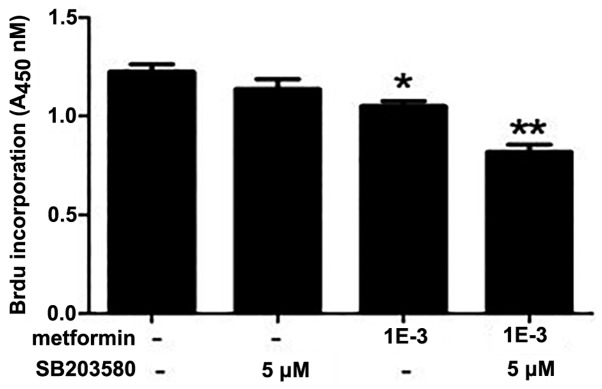
Metformin and p38 mitogen activated protein kinase (MAPK) SB203580 improved the sensitivity of SKOV3/DDP cisplatin-resistant ovarian cancer cells to cisplatin. The cells were treated with 2 μM cisplatin, followed by metformin and SB203580 treatment at different concentrations for 72 h. Cell proliferation was determined using the bromodeoxyuridine (BrdU) ELISA kit. Untreated cells are denoted by -. The results represent the means ± standard error of triplicate samples. Three independent experiments were performed. ^*^P<0.05, ^**^P<0.01, one-way analysis of variance.

Treatment with metformin and SB203580 alone significantly inhibited the proliferation of SKOV3/DDP cells, as compared with the control (P<0.05). Treatment of the cells with metformin combined with SB203580, synergistically inhibited cell proliferation, resulting in a statistically significant reduction in proliferation, as compared with both the control and the separate treatment strategies (P<0.01) (Fig. 5).

## Discussion

Epithelial ovarian cancer (EOC) is associated with the highest mortality rate of all known gynecological cancers, and surgery and chemotherapy are considered the gold standard of advanced EOC treatment. The occurrence of resistance to chemotherapy has a serious effect on the prognosis of ovarian cancer (1). The present study identified that the p38 MAPK signaling pathway was abnormally activated in ovarian cancer tissues that were resistant to platinum combined with paclitaxel, thus indicating that the MAPK signaling pathway may have an important role in chemoresistance. It was also observed that treatment with metformin inhibited the growth of drug-resistant ovarian cancer cells, and reversed drug resistance in cisplatin-resistant ovarian cancer cell lines. Previous studies have found that metformin inhibited the growth of ovarian cancer cells *in vitro* (8,9,16), which is consistent with the present findings.

Previous studies have shown that inhibitors of the mammalian target of rapamycin (mTOR) or the MAPK signaling pathways enhanced the anti-tumor effects of metformin. A suggested mechanism for this may be metformin enhancing the paclitaxel-induced cytotoxicity, as previously observed in non-small cell lung cancer cells (17). The present study found that metformin, in combination with the p38 MAPK inhibitor SB203580, improved cisplatin sensitivity in drug-resistant ovarian cancer cells. These results demonstrated that cisplatin chemoresistance may be associated with the MAPK signaling pathway in ovarian cancer. Tseng *et al* (17) found that metformin reduced the expression of the excision repair gene-1. The change in gene expression was shown to be mediated by phosphorylated MAPK pathways, which were being activated by paclitaxel. These findings suggest that inhibitors of p38 MAPK or the p42 MAPK signaling pathways, may increase the anti-tumor effects of metformin and paclitaxel.

Liu *et al* (18) found that a combination of the mTOR inhibitor RAD001 and metformin enhanced the cytotoxicity of chemotherapeutic drugs in breast cancer. Monteagudo *et al* (19) found that decreasing the expression of p42 MAPK through small interfering RNAs, significantly enhanced the anti-tumor effects of metformin in prostate cancer cells. These results suggest that metformin, in combination with signaling pathway inhibitors, may enhance the efficacy of platinum and paclitaxel in chemotherapy-resistant ovarian cancer (20). However, the precise mechanism is currently unclear. A previous study showed that metformin treatment reversed chemoresistance in breast cancer, and significantly inhibited multidrug resistance 1 (MDR1) gene transcription, which eventually significantly reduced the amount of the intracellular P-glycoprotein. It was demonstrated that metformin may reverse breast cancer chemoresistance by activating 5′ AMP-activated kinase, resulting in the suppression of MDR1 expression (21). The specific mechanisms underlying metformin reversal cisplatin-chemoresistance in ovarian cancer cells is a future research goal.

In conclusion, the MAPK signaling pathway in advanced EOC was shown to be abnormally activated in drug-resistant ovarian cancer tissues, as compared with primary ovarian cancer tissues. Metformin treatment improved the sensitivity of SKOV3/DDP cisplatin-resistant ovarian cancer cells, to cisplatin. There was a marked improvement in SKOV3/DDP cisplatin sensitivity, when the cells were treated with metformin in combination with the p38 MAPK inhibitor SB203580. The results of the present study support a potential clinical trial of metformin combined with MAPK signaling pathway inhibitors, in the treatment of chemotherapy-resistant ovarian cancers.

## References

[1] Morrison J, Haldar K, Kehoe S, Lawrie TA (2012). Chemotherapy versus surgery for initial treatment in advanced ovarian epithelial cancer. Cochrane Database Syst Rev.

[2] Ozols RF (2005). Treatment goals in ovarian cancer. Int J Gynecol Cancer.

[3] McLemore MR, Miaskowski C, Aouizerat BE, Chen LM, Dodd MJ (2009). Epidemiological and genetic factors associated with ovarian cancer. Cancer Nurs.

[4] Agarwal R, Kaye SB (2003). Ovarian cancer: strategies for overcoming resistance to chemotherapy. Nat Rev Cancer.

[5] National Comprehensive Cancer Network (2011). Clinical Practice Guidelines^™^ in Oncology (version 1.2011).

[6] Bodmer M, Becker C, Meier C, Jick SS, Meier CR (2011). Use of metformin and the risk of ovarian cancer: a case-control analysis. Gynecol Oncol.

[7] Romero IL, McCormick A, McEwen KA (2012). Relationship of type II diabetes and metformin use to ovarian cancer progression, survival, and chemosensitivity. Obstet Gynecol.

[8] Gotlieb WH, Saumet J, Beauchamp MC (2008). In vitro metformin anti-neoplastic activity in epithelial ovarian cancer. Gynecol Oncol.

[9] Rattan R, Graham RP, Maguire JL, Giri S, Shridhar V (2011). Metformin suppresses ovarian cancer growth and metastasis with enhancement of cisplatin cytotoxicity in vivo. Neoplasia.

[10] Iliopoulos D, Hirsch HA, Struhl K (2011). Metformin decreases the dose of chemotherapy for prolonging tumor remission in mouse xenografts involving multiple cancer cell types. Cancer Res.

[11] Rocha GZ, Dias MM, Ropelle ER (2011). Metformin amplifies chemotherapy-induced AMPK activation and antitumoral growth. Clin Cancer Res.

[12] Xing D, Orsulic S (2005). Modeling resistance to pathway-targeted therapy in ovarian cancer. Cell Cycle.

[13] Ohta T, Ohmichi M, Hayasaka T (2006). Inhibition of phosphatidylinositol 3-kinase increases efficacy of cisplatin in *in vivo* ovarian cancer models. Endocrinology.

[14] Lee S, Choi EJ, Jin C, Kim DH (2005). Activation of PI3K/Akt pathway by PTEN reduction and PIK3CA mRNA amplification contributes to cisplatin resistance in an ovarian cancer cell line. Gynecol Oncol.

[15] Kuo MT, Liu Z, Wei Y (2002). Induction of human MDR1 gene expression by 2-acetylaminofluorene is mediated by effectors of the phosphoinositide 3-kinase pathway that activate NF-kappaB signaling. Oncogene.

[16] Rattan R, Giri S, Hartmann LC, Shridhar V (2011). Metformin attenuates ovarian cancer cell growth in an AMP-kinase dispensable manner. J Cell Mol Med.

[17] Xie Y, Wang YL, Yu L (2011). Metformin promotes progesterone receptor expression via inhibition of mammalian target of rapamycin (mTOR) in endometrial cancer cells. J Steroid Biochem Mol Biol.

[18] Tseng SC, Huang YC, Chen HJ (2013). Metformin-mediated downregulation of p38 mitogen-activated protein kinase-dependent excision repair cross-complementing 1 decreases DNA repair capacity and sensitizes human lung cancer cells to paclitaxel. Biochem Pharmacol.

[19] Liu H, Scholz C, Zang C (2012). Metformin and the mTOR inhibitor everolimus (RAD001) sensitize breast cancer cells to the cytotoxic effect of chemotherapeutic drugs in vitro. Anticancer Res.

[20] Monteagudo S, Pérez-Martinez FC, Pérez-Carrión MD (2012). Inhibition of p42 MAPK using a nonviral vector-delivered siRNA potentiates the anti-tumor effect of metformin in prostate cancer cells. Nanomedicine (Lond.).

[21] Kim HG, Hien TT, Han EH (2011). Metformin inhibits P-glycoprotein expression via the NF-κB pathway and CRE transcriptional activity through AMPK activation. Br J Pharmacol.

